# In Situ Quantification of Polyhydroxybutyrate in Photobioreactor Cultivations of *Synechocystis* sp. Using an Ultrasound-Enhanced ATR-FTIR Spectroscopy Probe

**DOI:** 10.3390/bioengineering8090129

**Published:** 2021-09-21

**Authors:** Philipp Doppler, Christoph Gasser, Ricarda Kriechbaum, Ardita Ferizi, Oliver Spadiut

**Affiliations:** 1 Research Division Biochemical Engineering, Institute of Chemical, Environmental and Bioscience Engineering, TU Wien, Gumpendorfer Strasse 1a, 1060 Vienna, Austria; philipp.doppler@tuwien.ac.at (P.D.); ricarda.kriechbaum@tuwien.ac.at (R.K.); 2 usePAT GmbH, Schönbrunner Strasse 231/2.01, 1120 Vienna, Austria; christoph.gasser@usepat.com (C.G.); ardita.ferizi@usepat.com (A.F.)

**Keywords:** polyhydroxyalkanoates, PHB, PAT, *Synechocystis* sp. PCC 6714, process monitoring, ultrasound particle manipulation

## Abstract

Polyhydroxybutyrate (PHB) is a very promising alternative to most petroleum-based plastics with the huge advantage of biodegradability. Biotechnological production processes utilizing cyanobacteria as sustainable source of PHB require fast in situ process analytical technology (PAT) tools for sophisticated process monitoring. Spectroscopic probes supported by ultrasound particle traps provide a powerful technology for in-line, nondestructive, and real-time process analytics in photobioreactors. This work shows the great potential of using ultrasound particle manipulation to improve spectroscopic attenuated total reflection Fourier-transformed mid-infrared (ATR-FTIR) spectra as a monitoring tool for PHB production processes in photobioreactors.

## 1. Introduction

Every day, huge quantities of petroleum-based plastics are produced. The majority is for single-use purposes, not recycled and not biodegradable. Their production and disposal by incineration releases high amounts of the greenhouse gas carbon dioxide (CO_2_) into the atmosphere. Nonbiodegradable plastics carelessly disposed in nature are polluting almost all habitats on earth. Plastic slowly breaks down into microplastics, threatening life in water and on land [1]. In times of serious climate change, the Paris Agreement on limiting global warming, the single-use plastics ban of the European Union, and other initiatives to avoid accumulation of plastics all over the planet, research on new technologies and materials is essential [2]. These developments should help to satisfy the Sustainable Development Goals (SDGs) proposed by the United Nations for a more sustainable future of human society [3].

One possible solution to simultaneously reduce the atmospheric CO_2_ concentration and generate biodegradable plastics are cyanobacteria. Several species of cyanobacteria produce different types of polyhydroxyalkanoates (PHAs) photoautotrophically, i.e., solely out of light energy and CO_2_ [4]. PHAs are alternatives to most petroleum-based plastics, and they are fully biodegradable [5]. An important representative is polyhydroxybutyrate (PHB). Alongside glycogen, PHB is accumulated by some cyanobacterial species as carbon and energy storage compound [6,7,8]. It is produced during nutrient starvation (nitrogen or phosphorus) or other stress-inducing factors [9,10].

The reason these bioplastics from cyanobacteria did not find their way into applications yet is that scaling-up the production processes is challenging. Usually, externally illuminated glass reactors with a high surface-to-volume ratio are used [7,11,12]. For every step during scale-up, the availability of CO_2_, nutrients, and most importantly, light is different, and biomass growth and PHB productivity strongly vary [13]. Thus, in situ quantification tools used as process analytical technology (PAT) to determine the PHB content in real-time are inevitable. Karmann et al. utilized flow cytometry for at-line PHB quantification [14], and Gutschmann et al. investigated the use of photon density wave spectroscopy for in-line PHB monitoring [15]. Vibrational spectroscopy represents another powerful technique for probing liquid as well as solid samples in a nondestructive manner, and it is also capable of in-line operation. Attenuated total reflection Fourier-transformed mid-infrared (ATR-FTIR) spectroscopy can be used to quantify the amount of PHB or glycogen accumulated within the cell [16].

In a typical in-line ATR probe, light in the mid-IR region from 400–4000 cm^−1^ is totally reflected within the diamond tip. This results in an evanescent field that penetrates approximately 1–2 μm of the cultivation broth and interacts with all molecules [17]. The changes in light intensities are recorded, and the spectra can be evaluated by univariate calibration or chemometric tools [16,18]. ATR-FTIR instruments are commercially available, and applications for in-line, on-line, or at-line monitoring are established PAT tools, e.g., for *P. chrysogenum* or *E. coli* processes [19,20,21]. Further, ATR-FTIR studies were done for characterization of PHAs [22], and for PHB production processes using bacteria [23,24,25]. In general, a specific cell spectrum in liquid culture is only achievable if the evanescent field is mainly populated by cells. Usually in stirred cultivation broths, only the liquid constituents are accessible. Different strategies were employed to selectively enhance the signal attributed to the cells, e.g., using an on-line loop to bring medium through a flow cell equipped with an ATR-element. By selectively stopping the flow, cells settle on the ATR element and are then accessible for analysis with ATR-FTIR spectroscopy. This was shown for quantification of PHB in *E. coli* [24]. After the measurement, the cells are resuspended as flow is re-established. This prolonged procedure could influence the cells due to oxygen or nutrient limitations, as well as produce biofilms as cells are not efficiently resuspended. To avoid these effects in biotechnological processes ultrasound (US) particle manipulation devices can be used [26].

It is, however, desirable to have a fast in-line measurement of the PHB content for process monitoring and control. By using spectroscopic probes and acoustic traps, which can perform in-line particle (cell) manipulation, cells can be directly accumulated in the evanescent field of an ATR probe, making them available for measurement. These acoustic traps use US standing wave fields to trap cells in the fermentation broth [27]. By alternating US frequencies, the acoustic pressure nodes carrying the cells can be moved and the cells are pushed onto the ATR diamond for direct acquisition of IR cell spectra. US particle manipulation in combination with an ATR-FTIR probe was successfully used to generate cell spectra containing quantitative information of accumulated carbohydrates in *S. cerevisiae* cultivations [28,29].

Here, we present a US particle manipulation device to enhance the quality of in situ ATR-FTIR spectra of *Synechocystis* sp. PCC 6714 during PHB accumulation. It was directly inserted into the bioreactor, enabling in-line measurement of PHB and glycogen content of the cyanobacterial cells. We did photobioreactor cultivations to evaluate the applicability of the technology as a real-time PAT tool for monitoring, and to avoid time-consuming and laborious PHB off-line analytics involving toxic chemicals.

## 2. Materials and Methods

### 2.1. Strain, Media, and Preculture Preparation

An axenic culture of wild-type *Synechocystis* sp. PCC 6714 accumulating PHB under nitrogen-limiting conditions was used [30,31]. The precultures for inoculation of photobioreactor cultivations were grown in 250 mL Erlenmeyer flaks containing 50 mL BG11 medium with 1.5 g·L^−1^ NaNO_3_ [32]. The cells were incubated in a Minitron incubation shaker (Infors, Basel, Switzerland) at 28 °C, 150 rpm (25 mm amplitude) with 3% CO_2_ atmosphere under continuous illumination of 50 μmol·m^−2^·s^−1^.

### 2.2. Bioreactor Cultivations

For photobioreactor experiments a Ralf bioreactor system (Bioengineering, Wald, Switzerland) with 6.7 L total volume respectively 5.4 L working volume was equipped with two 65 mm turbine impellers, and LED strips for external, continuous illumination via the glass jacket. 10 m of warm-white LED stripes (Paulmann, Völksen, Germany) carrying 200 LEDs with a total light power of 1,920 lm were uniformly wrapped around the vessel. The inner diameter of the reactor was 148 mm, and the illumination provided in the center, measured in cell-free BG11 medium, was 250 μmol·m^−2^·s^−1^. pH was measured in-line via an EasyFerm Plus pH probe (Hamilton, Bonaduz, Switzerland). Pressurized air and CO_2_ were separately controlled by two type 4850 mass flow controllers (Brooks Instruments, Hatfield, PA, USA) operated by the 0254 control unit (Brooks Instruments, Hatfield, PA, USA).

5.4 L BG11 medium containing approx. 750 mg·L^−1^ NaNO_3_ was filled into the vessel prior to autoclaving [32]. Aeration flow of the 5% CO_2_-enriched air was in total 270 mL·min^−1^ (0.05 vvm). The pH was controlled at 8.5 ± 0.05 via automatic addition of 2 N KOH throughout the whole process. Temperature was set to 28 °C and the stirring speed to 180 rpm. For inoculation, the preculture was added via septum to a final optical density at 750 nm (OD_750_) of 0.12. Samples were drawn in regular intervals every day until the end of process. To monitor cell growth OD_750_ was determined for every sample in 1 mL cuvettes on a Nanodrop One photometer (Thermo Fisher Scientific, Waltham, MA, USA). Dry cell weight (DCW) was estimated via following correlation found in previous studies [33]:

DCW [mg·L^−1^] = 168.9 ∗ OD_750_ (1)

Nitrate concentrations in the medium were determined on an ICS-6000 ion chromatography system with an IonPac AS11 column (Thermo Fisher Scientific, Waltham, MA, USA). The method was recently described in detail elsewhere [34].

### 2.3. In-Line Ultrasound-Enhanced ATR-FTIR Measurements

A ReactIR 45m spectrometer with plug-and-play interface was connected to a 1.5 m silver halide optical fiber ATR probe with diamond tip (Mettler Toledo, Columbus, OH, USA). It was used for collecting mid-IR spectra between 800 and 1,800 cm^−1^ at a resolution of 4 cm^−1^. The detector was constantly cooled by liquid nitrogen and the device purged with dry air. For data acquisition, the software iC IR 4.2 (Mettler Toledo, Columbus, OH, USA) was used. As a background, cultivation medium before inoculation was measured. A spectrum was recorded every minute with 256 scans.

**Scheme 1:**
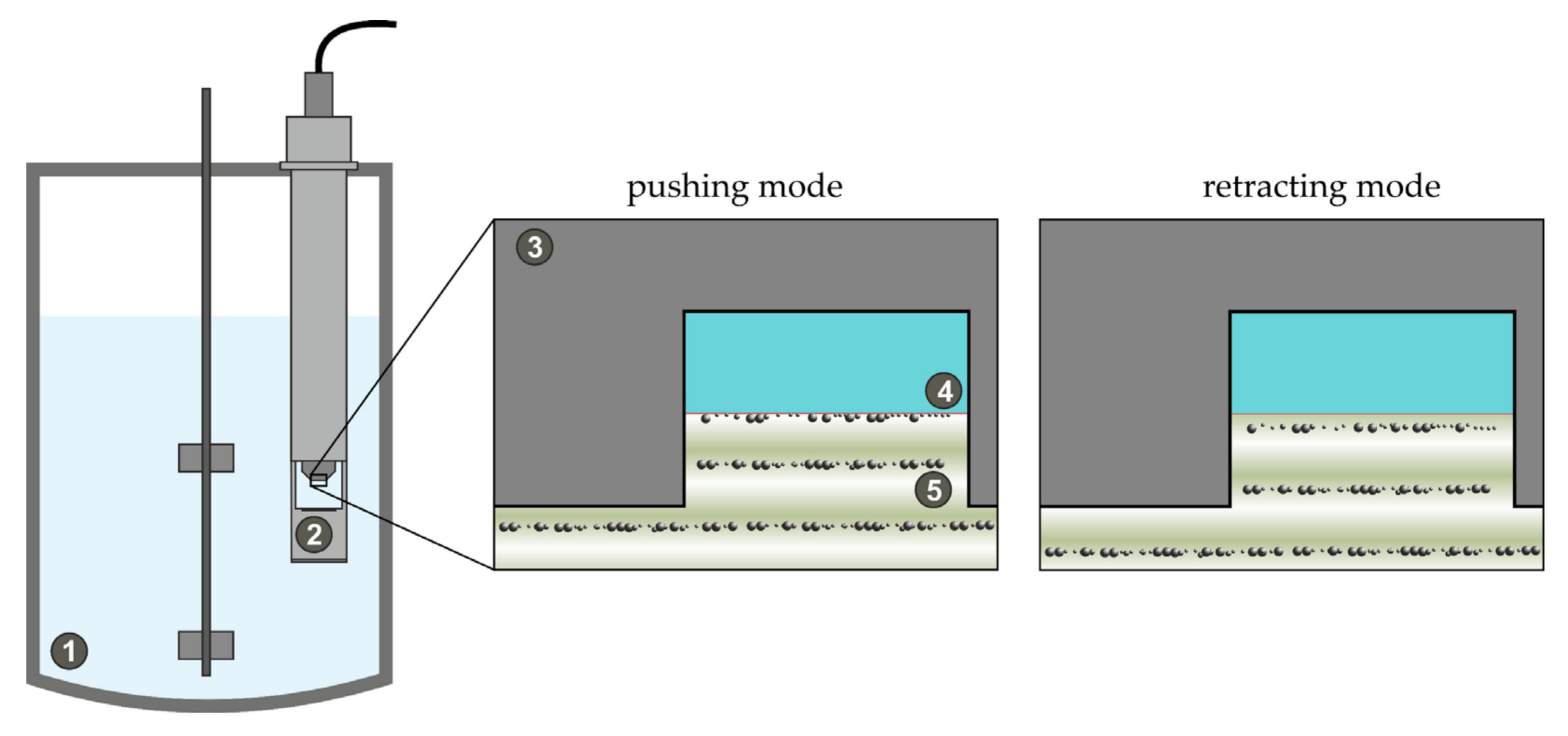
Experimental setup: (1) a glass bioreactor with stirrer. (2) The sonic-catch add-on in combination with the ReactIR 45m diamond ATR probe formed the US trap. (3) Detail of ATR probe tip (4), where the US standing wave field (shaded in green, (5)) catches cells in pressure nodal points. By accurate control of this US field, cells can either be pushed into evanescent field of ATR or retracted from it.

The ATR probe head together with the soniccatch add-on (usePAT GmbH, Vienna, Austria) formed a US trap (see Scheme 1). The created standing wave US field held cells in place directly in the reactor. By accurate control of the field parameters via the US transducer through the US amplifier sonicamp (usePAT GmbH, Vienna, Austria), the cells could either be pushed into the evanescent field of the ATR or retracted from it, enabling a selective in-situ FTIR measurement of the cells and their PHB and glycogen content. The distance of ATR surface to US transducer was 2.8 mm, the frequency of the driving amplifier was set to 2.18 MHz. The used trapping protocol involved four steps: firstly, cells were trapped at a catching frequency, followed by a successive stepwise ramp to push the cells into the evanescent field. Afterwards, the frequency was adjusted back to retract the cells. Finally, the cells were released, and the cycle started anew. This ensured the catching and measurement of new cells from the fermentation broth.

Spectra treatment and evaluation was done in Python 3.8 using the numpy, scipy, and pandas libraries. Spectra were smoothed using a Savitzky–Golay filter with a window length of 15 and a third order polynomial. Catching of cells was verified by evaluating the integrated intensity of the Amide II band (ca. 1540 cm^−1^), since this absorption band is characteristic of the cells. Pristine cell spectra were obtained by subtracting consecutive spectra of retracted and pushed cells, leading to drift-free absorbance spectra. Since the amount of caught and pushed cells varied, for the determination of PHB and glycogen content the spectra were normalized to the Amide II band. For PHB quantification, the most important band at 1738 cm^−1^, corresponding to the carbonyl stretch, was integrated, and correlated to the amount of PHB in the cells determined by the reference method. Since this band is not overlapped by any other substance in the matrix, a simple univariate calibration was possible. The determination of glycogen was performed analogously using the absorption band at 1030 cm^−1^.

### 2.4. Off-Line Quantification of PHB and Glycogen

After centrifugation of 1 mL culture aliquots, the cell pellets were dried at 75 °C for 24 h, and directly used for PHB and glycogen sample preparation and measurement [33]. Briefly, PHB was quantified on an UltiMate 3000 HPLC system (Thermo Fisher Scientific, Waltham, MA, USA) with an Aminex HPX-87 H column (Bio-Rad Laboratories, Hercules, CA, USA). Glycogen was determined on an ICS-6000 ion chromatography system with a CarboPac PA10 column (Thermo Fisher Scientific, Waltham, MA, USA).

## 3. Results

### 3.1. Photobioreactor Cultivations

**Figure 1:**
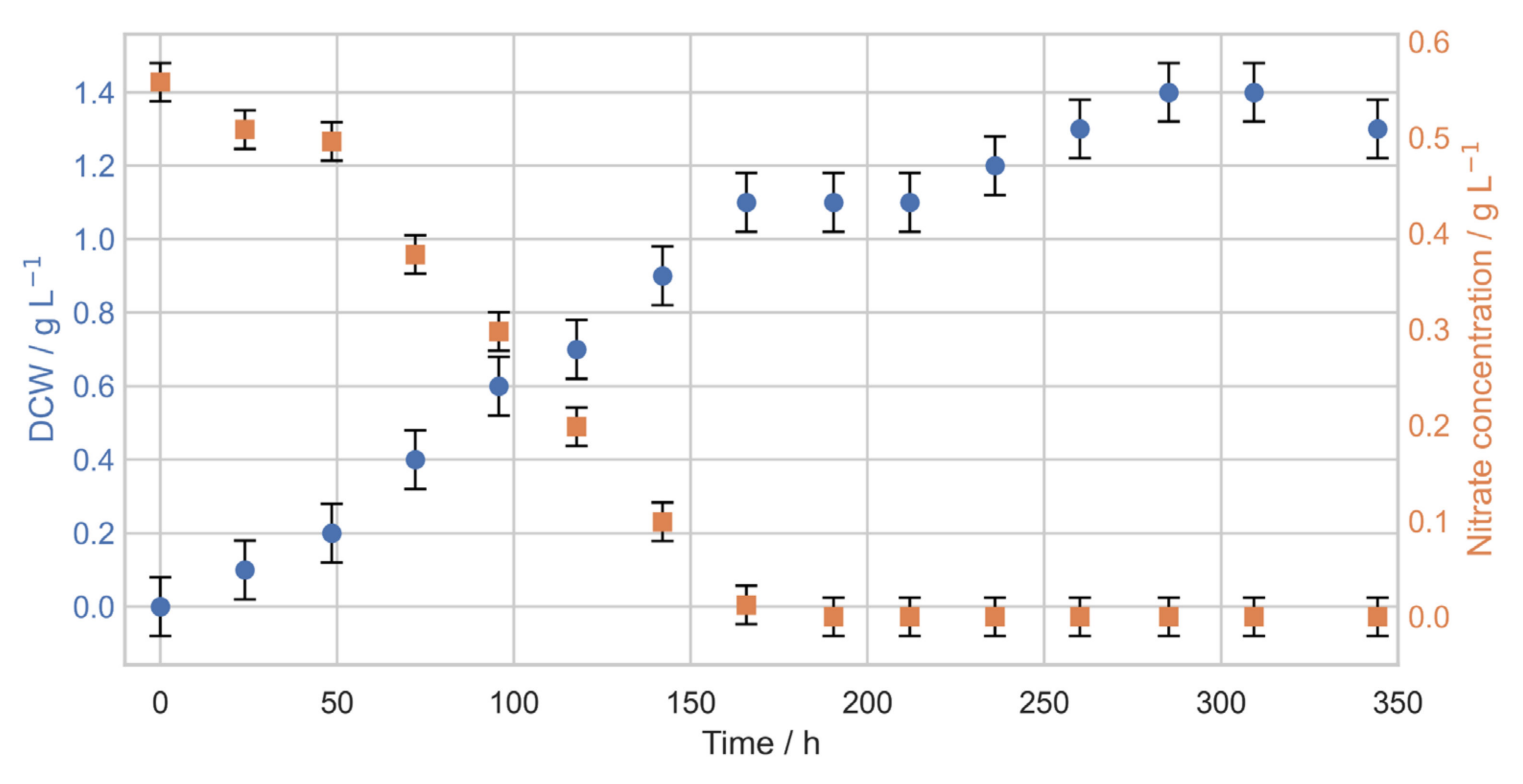
A photobioreactor cultivation of Synechocystis sp. PCC 6714 under nitrogen-limiting conditions showing the DCW (blue) and nitrate concentration (orange) during process. Error bars reflect the error of calibration curve.

For accumulation of PHB within the wild-type *Synechocystis* sp. PCC 6714 strain photobioreactor cultivations were conducted, and biomass growth and nitrate content monitored (Figure 1).

During the biomass growth phase, growth was almost linear within the first seven days (168 h) with an average growth rate μ_linear_ = 0.15 g·L^−1^·d^−1^ to a value of 1.06 g·L^−1^. The available nitrate in the medium of 9.0 mM was consumed after 168 h, which resulted in an average biomass per spent nitrate yield of 1.87 g·g^−1^. Following the depletion of nitrogen, PHB production started. After a maximum of 1.39 g·L^−1^ biomass was reached at 309 h, the cell concentration started to decline, and thus, the process was stopped at a final DCW of 1.33 g·L^−1^ after 344 h (Figure 1).

### 3.2. Ultrasound-Enhanced ATR-FTIR Spectra

**Figure 2:**
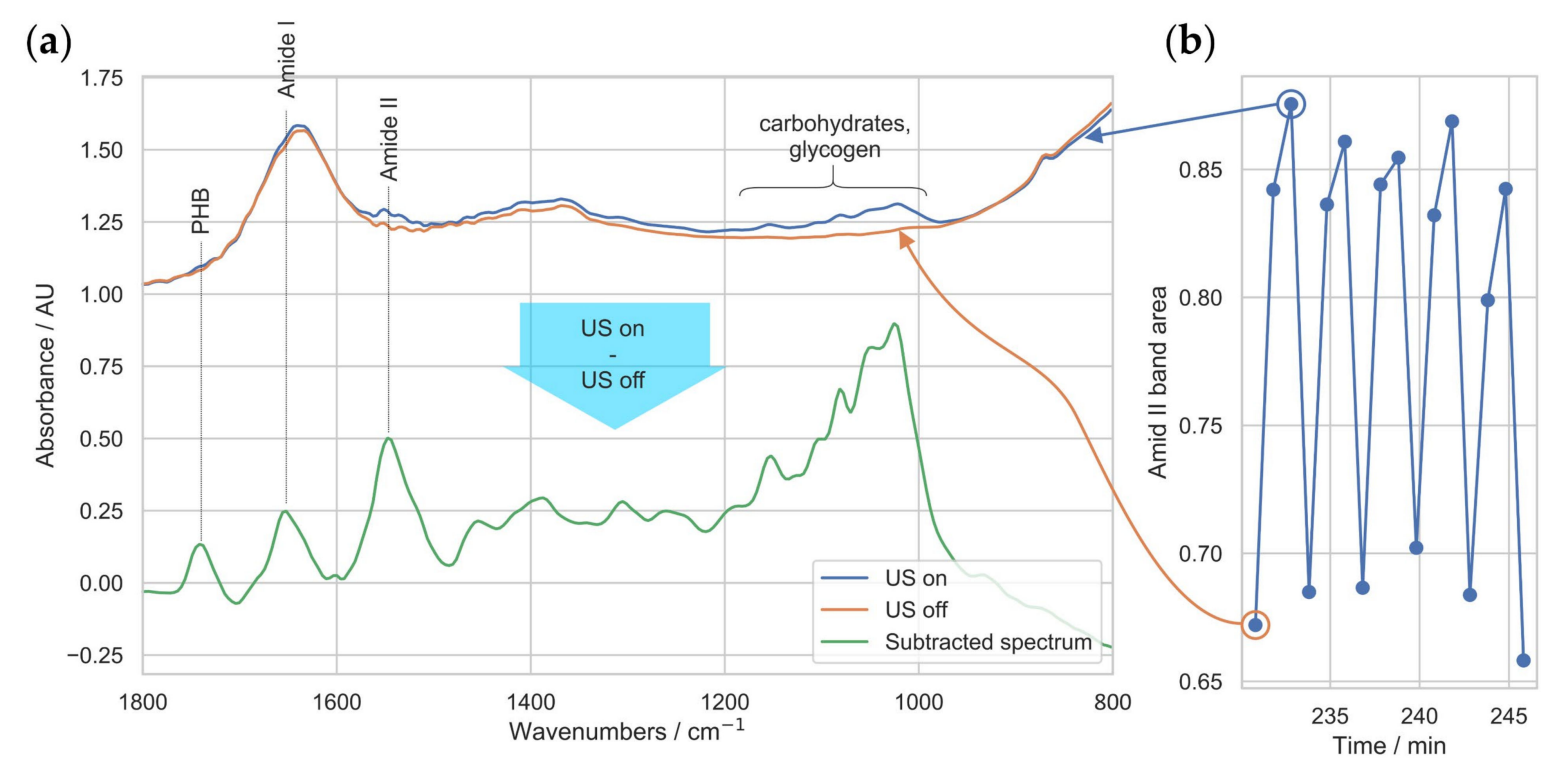
Typical process FTIR spectra. (**a**) Two selected spectra with activated (blue) and deactivated US capture (orange) are depicted on top. Subtraction yielded pure spectra of *Synechocystis* sp. 6714, shown at bottom (green). (**b**) Integrated signal from the Amide II band, showing modulation occurring when US trap was in pushing mode vs. retracting mode.

Figure 2a depicts typical process FTIR spectra, with the orange spectrum at the top representing a spectrum in retracted state and the spectrum in blue representing the pushing state. Clear bands attributed to specific vibrational transitions in organic material, and in this case, cells can be seen in the blue spectrum, e.g., the Amide I band at 1650 cm^−1^, the Amide II band at 1540 cm^−1^, or the fingerprint region from 990–1200 cm^−1^. Figure 2b shows the integrated intensity of the Amide II band at 1540 cm^−1^. When the cells were pushed into the evanescent field of the ATR, the intensity raised, and when the cells were pulled out again, it decreased. The resulting modulation was stable and allowed for the subtraction of a pair of differently manipulated spectra, which yielded a pristine spectrum of the cells, shown at the bottom of Figure 2a in green. As indicated in Figure 2b, the intensity of the Amide II band was used to automatically detect pairs for spectra subtraction, as it was not affected as strongly by shifts of the OH-deformation band of water at 1650 cm^−1^ compared to that of the Amide I band.

**Figure 3:**
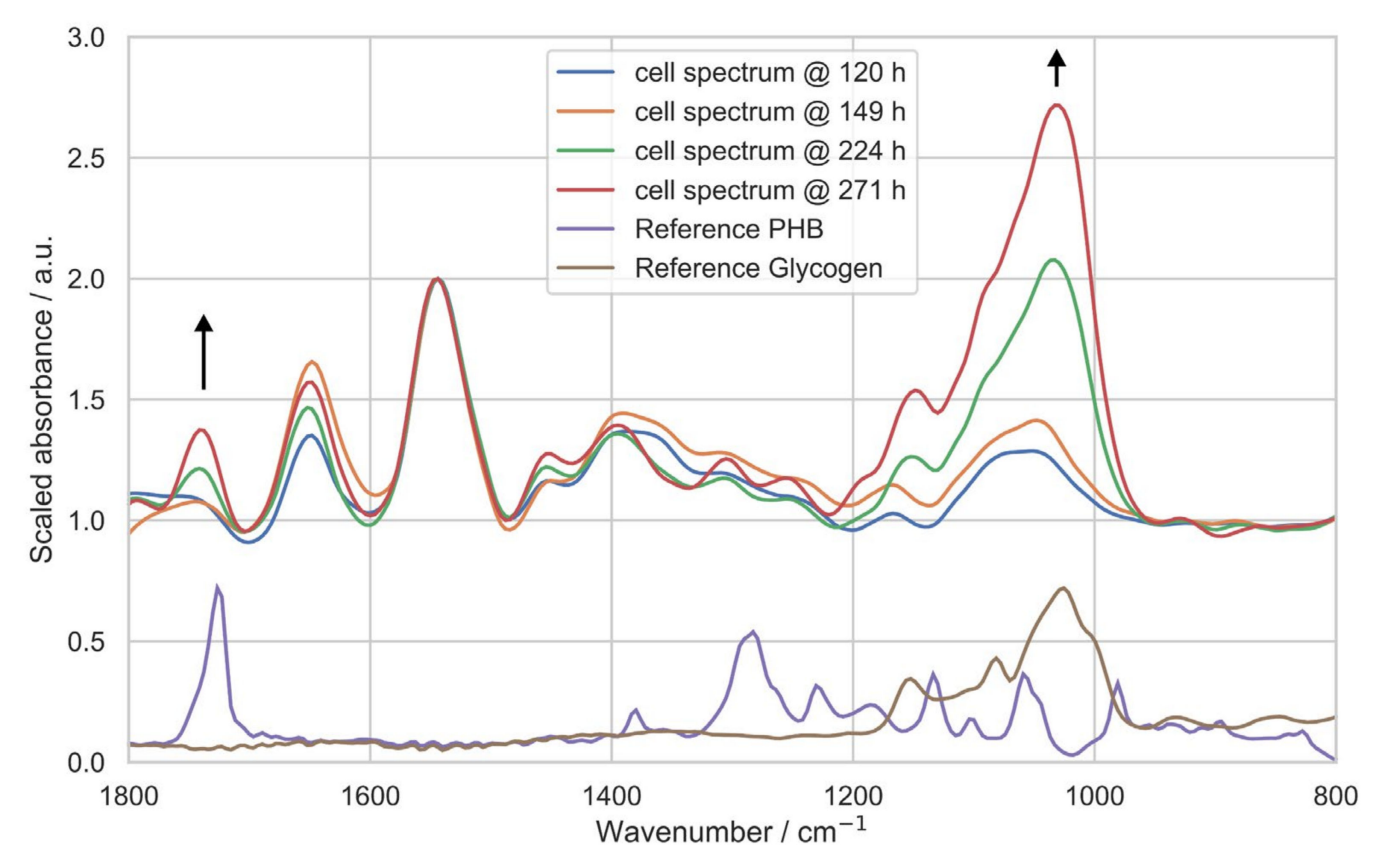
Top: in-line spectra of cells during different times in fermentation. PHB and glycogen bands were increasing steadily. Bottom: reference spectra of dry PHB and glycogen powder, showing approximate position of specific absorption bands.

In these subtracted spectra, the accumulation of PHB in the cell during the nitrogen deprived cultivation became clearly visible, as the band attributed to the carbonyl stretch of the PHB at 1738 cm^−1^ was free standing and could thus be used directly for determination of the PHB content. It was in good agreement with the reference spectrum obtained by direct measurement of PHB powder (Figure 3).

### 3.3. In Situ Quantification of PHB Content

Cell spectra obtained during the cultivations revealed a steady increase in the intensity of the PHB absorption band at 1738 cm^−1^, as shown in Figure 3. These spectra were normalized to the Amide II band to accommodate the fact that during catching of new cells, not the exact same amount of material could be caught and was pushed into the evanescent field of the ATR for FTIR measurement. In this case, we used nonoverlapping bands as an internal standard for intensity correction, and the Amide II band was not affected by either PHB or glycogen, or other components of the cultivation broth. For calibration, reference values were needed. For the characterization of the cultivation at different time intervals, offline samples were taken and analyzed for PHB and glycogen content. Because the timestamp of the offline reference samples and the in-line measurements did not necessarily coincide, the offline references were used to fit a model describing its evolvement over time. With the obtained reference points, a univariate calibration (band integral at 1738 cm^−1^ for PHB, band height at 1025 cm^−1^ for glycogen) was performed. This resulted in coefficients of determination (R^2^) and limit of quantifications (LOQ) of 0.91 and 18.3 mg·g^−1^ and 0.90 and 71.2 mg·g^−1^ for PHB and glycogen, respectively, as summarized in Table 1.

**Figure 4:**
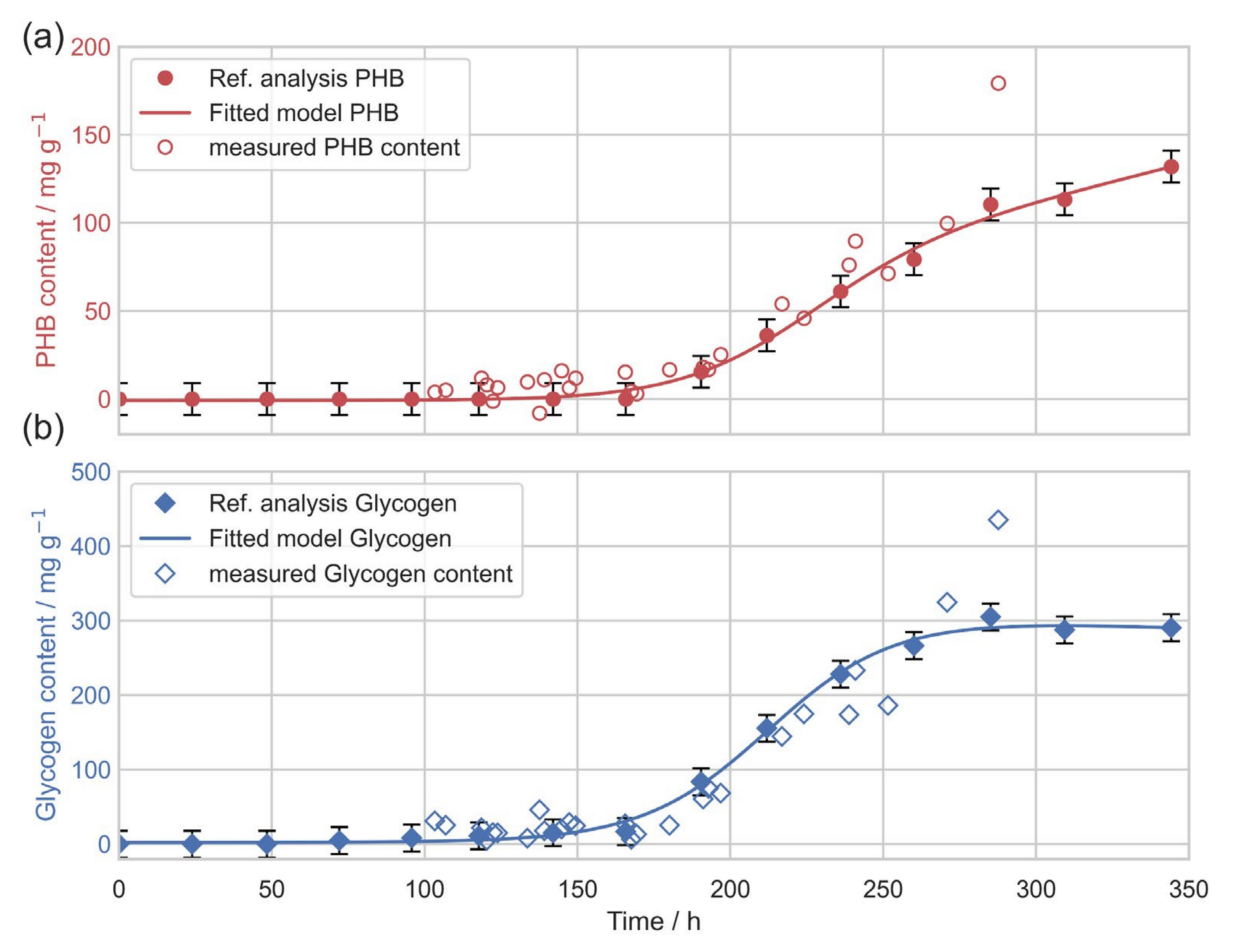
Trend of accumulated storage compounds during *Synechocystis* sp. PCC 6714 cultivation. (**a**) In-cell PHB; (**b**) glycogen content. Reference analysis was performed offline (filled markers). Profile was modeled to extracted reference points for caught spectra. In-line measured quantities are depicted as unfilled markers. Error bars reflect error of calibration curve.

With an established calibration, the trends of accumulated PHB and glycogen during the cultivation were plotted (Figure 4). The in-line determined PHB and glycogen content were in good agreement with the references obtained from offline analytics.

## 4. Discussion

For the production of PHB classical two-phase cultivation processes were performed. During the first stage, the biomass growth phase, linear growth with an average rate of 0.15 g·L^−1^·d^−1^ was observed until 168 h of process time. Linear photoautotrophic growth is typical for stirred photobioreactors since the cells are usually light-limited [12]. This is due to the long distances photons have to travel in the wide vessel, and it occurs even in diluted cultures [35,36]. After 168 h, the nitrate was completely consumed. This initiated the PHB production phase to a maximum intracellular product content of 132 mg·g^−1^ (13.2%) of DCW, respectively a volumetric PHB concentration of 175 mg·L^−1^, which is in the range of published values for this strain [30]. The process was stopped after biomass concentration started to decline after 344 h.

Throughout the cultivation, the frequency of the US trap was alternated according to the proposed four-step protocol for trapping, pushing, retracting and releasing. By this, different ATR-FTIR spectra were obtained, which yielded pristine spectra of caught *Synechocystis* sp. PCC 6714 cells. Specific bands for PHB at 1738 cm^−1^ (C-O stretch) and the characteristic region for glycogen between 950 and 1200 cm^−1^ (combinations of C-O, C-C and C-O-H stretch) could be assigned by comparison to that of off-line spectra of respective pure standard material and literature values [37,38]. The shift between measured in-line spectra of PHB compared to that of pure, dry PHB powder was ascribed to different IR absorption maxima of amorphous and crystalline PHB. Thus, the PHB accumulated within *Synechocystis* sp. PCC 6714 was predominantly amorphous [24]. After correlating off-line to in situ acquired spectral data, LOQ values for cellular PHB and glycogen content of 18.3 and 71.2 mg·g^−1^ were calculated. The possibility to directly measure PHB in the cell by simply using the characteristic band intensity of PHB is a straightforward univariate analysis technique, void of complex chemometric modelling. This allows for straight interpretation of the acquired spectra.

We successfully utilized a US particle manipulation device to enhance ATR-FTIR spectra quality for PHB and glycogen quantification in situ in a photobioreactor. The accumulation of these compounds was quantified and monitored throughout the cultivation process. This work shows the great potential of this method as a real-time PAT monitoring tool for PHB production processes.

## Figures and Tables

**Table 1:** Calibration results for PHB and glycogen in situ quantification.

Analyte	Method	Adj. R^2^ [–]	LOQ [mg·g^−1^]
PHB	Band integration (1705–1774 cm^−1^), baseline corrected	0.91	18.3
Glycogen	Band height at 1025 cm^−1^, baseline corrected	0.90	71.2

## Data Availability

The data presented in this study are available on request from the corresponding author.
